# Modeling and Optimization of the Creep Behavior of Multicomponent Copolymer Nanocomposites

**DOI:** 10.3390/s23031190

**Published:** 2023-01-20

**Authors:** Gangping Bi, Bowen Xiao, Yuanchang Lin, Shaoqiu Yan, Shuge Li, Ying Tang, Guotian He

**Affiliations:** 1 Chongqing Institute of Green Intelligent Technology, Chinese Academy of Sciences, Chongqing 400714, China; 2 College of Mechanical Engineering, Chongqing University of Technology, Chongqing 400054, China; 3 Chongqing Key Laboratory of Artificial Intelligence and Service Robot Control Technology, Chongqing Institute of Green Intelligent Technology, Chinese Academy of Sciences, Chongqing 400714, China; 4 College of Artificial Intelligence, Chongqing School, University of Chinese Academy of Sciences, Chongqing 400020, China

**Keywords:** composite filling, resistance creep model, tactile sensing, graphene, Ni

## Abstract

Polymer creep can significantly reduce the safety and dependability of composite applications, restricting their development and use in additional fields. In this study, single-factor and multi-factor analysis techniques were employed to systematically explore the impacts of nickel powder and graphene on the resistive creep of sensing units. The creep model between the rate of resistance changes and the pressure was established, and the material ratio was optimized to obtain a high creep resistance. The results demonstrated that the creep resistance was best when the filling particle was 10 wt.% and the ratio of nickel powder to graphene was 4:21, which was approximately 60% and 45% lower than the filling alone and the composite filling before optimization, respectively; the R^2^ of the theoretical value of the resistance creep model and the experimental value of the creep before and after optimization was 0.9736 and 0.9812, indicating that the resistance creep model was highly accurate. Consequently, the addition of filler particles with acceptable proportions, varied shapes, and different characteristics to polymers can effectively reduce polymer creep and has significant potential for the manufacture of sensing units for tactile sensors.

## 1. Introduction

Polymer nanocomposites are multiphase solid materials including at least one nanoscale component, and their unusual features have inspired numerous researchers to conduct substantial and in-depth research on them [1]. In the realm of flexible haptic sensors, carbon-based [2,3,4] and metal-based [5,6,7] conducting polymer nanoparticles [8] have been widely utilized. Creep is one of the most fundamental expressions of the static viscoelasticity of polymer materials, which is a type of material failure [9] and one of the most significant barriers to the application of polymer nanocomposites in the field of haptic sensors. Creep is a phenomenon in which the distortion of a material changes over time in the presence of a constant external environment [10]. Thus, under the influence of a specific temperature and a constant external force, the deformation of a material varies constantly with time. The primary cause for this is that the molecular bonds between polymer chains are loosely linked, resulting in slipping and tugging when a consistent force is applied. When the applied force is strong enough, it causes molecular chains to separate and recombine. When the external force is removed, residual creep occurs when a small portion of the molecular chain is unable to return to its previous condition due to the violent movement. Therefore, strengthening the creep resistance of polymers by enhancing their connections is a hot topic in contemporary research [11], which has significant consequences for the creation of touch sensors.

The recent nanocomposite study demonstrated that the volume percentage of particles, particle size, the ratio of silicone rubber to silicone oil, the amount of applied pressure, the pressure holding time, and temperature, among others, are the primary factors influencing creep. Fan et al. [12] examined the impacts of particle volume fraction, particle size, etc., on sensor creep and determined that the resistance to sensor creep reduces as the number of contributing factors other than particle size increases. Ayesha Naz et al. [13] investigated the effect of the hydrothermal reduction of graphene oxide (RGO) on the creep of polypropylene matrix and concluded that polymer nanocomposites generated by high-temperature polymerization can significantly improve creep resistance; at the same time, the effect of carbon nanotubes and graphene polymers on creep under the same matrix was also investigated separately, and the comparison revealed that graphene has better creep resistance than carbon nanotubes. Several studies have also examined the inclusion of nano clay [14], carbon nanotubes [15], and nanoparticles [16] in the polymer matrix, which drastically altered the polymer’s creep properties. Graphene possesses a huge specific surface area and exceptional mechanical characteristics [1]. In comparison to CB (carbon black)/PS (polystyrene) and CNT (carbon nanotubes)/PS nano-composites, graphene/PS nano-composites demonstrate superior creep resistance, according to Tang et al. [11]. Silicone rubber (SR) has been widely employed as a typical engineering polymer material [17] due to its low modulus, high elasticity, flexibility, and excellent environmental resilience. Most importantly, silicone rubber is soft, stable, heat resistant, non-toxic, and tasteless [18], making it an ideal material for laboratory research. Furthermore, silicone rubber and graphene, both lipophilic materials, can disperse graphene uniformly, providing a good solution to the problem of graphene agglomeration [19]. In terms of sensor theory, Mizera Č et al. [20] studied the creep behavior of fibers and proposed a two-branch generalized Maxwell model and a Kelvin model to describe the relaxation and creep behavior of composite fibers. Cholleti E R et al. [21] investigated the creep behavior of silicone elastomer composites and used a second-order generalized Kelvin–Voigt model to explain the experimental phenomena and a feature-fitting approach to determine the material coefficients. Maria H J et al. [22] studied nanoparticle-doped polymers using the stretched-exponential Kohlrausch equation and Maxwell-Weichert model, which well explained the rearrangement phenomenon of polymer chains. Xia et al. [23] proposed a homogenization scheme for strain considering the volume change and strain-dependent electron tunneling of nanocomposites, which well explained the strain dependence of composite resistance. Similarly in tension and compression, Xia et al. [24] revealed the difference between the tensile and compressive sensing performance of the sensor by establishing an electromechanical coupling homogenization scheme. In conclusion, these findings focused mostly on the effect of single-component filler materials on creep, whereas very few multi-component filler materials have been described.

In this study, the effects of the mass fraction of filler particles and the ratio of two different filled particles on the creep resistance of polymers were primarily explored using single- and multi-factor analysis. Firstly, we proposed a polymer creep resistance model and performed a qualitative analysis; secondly, we investigated the effects of nickel powder and graphene on the creep resistance of the polymer separately; again, we studied the effects of the ratio of nickel powder and graphene on the creep resistance of the polymer under the assumption of maintaining a certain mass fraction. Thirdly, we investigated the effect of different mass fractions of filled particles on the creep resistance of the polymer; finally, we realized the formulation of sensing units with good creep resistance by optimizing the response surface, compared the creep magnitude before and after optimization, and compared the experimental creep values with the theoretical values to verify the accuracy of the model.

## 2. Materials and Methods

### 2.1. Materials

The raw materials required for this formula include silicone rubber matrix (purchased from Shenzhen Red Leaf Technology Co., Ltd. (Shenzhen, China) produced two-component room temperature vulcanization silicone rubber RTV-2), filled particles of 500 nm nickel powder (Zhong ye Xin dun alloy) and multilayer graphene (Suzhou Carbon Feng purity 95%, thickness 3.4–8 nm, the number of layers 5–10 layers), silicone oil using ordinary dimethyl silicone oil, coupling agent (using Guangzhou Long kai Chemical Co., Ltd., Guangzhou, China, silane coupling agent KH550), and curing agent (107 types of curing agent produced by Shenzhen Red Leaf Technology Co., Ltd.).

### 2.2. Preparation of Samples and Methodology

**Figure 1:**
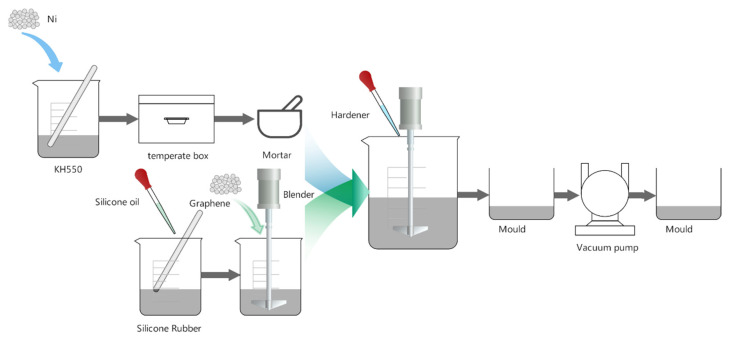
Sensor unit preparation process.

The following steps comprise the preparation procedure: First, the corresponding components of various materials were weighed with an electronic balance, and the surface of the nickel (Ni) nanopowder was pretreated with a coupling agent, put into a thermostat for drying, and then ground into a powder after treatment; secondly, silicone rubber (SR) and silicone oil were mixed in the appropriate proportions and stirred to create a homogeneous mixture; then, multilayer graphene (MLG) was added to the homogeneous silicone rubber and silicone oil mixture Then, the pre-treated nickel nanopowder was added to the mixture for thorough stirring, followed by the addition of the appropriate curing agent and continued stirring; the mixture was poured into the mold, and the defoaming step was performed in the vacuum pump. Finally, the defoamed mold was removed and placed at room temperature for molding. Figure 1 illustrates the experimental method.

To investigate the influence of nickel powder and graphene on the resistance to creep of polymeric materials, five sets of experiments were designed, and the principal parameters of the five experimental formulations were given. Table 1 lists the specific experimental protocols.

**Figure 2:**
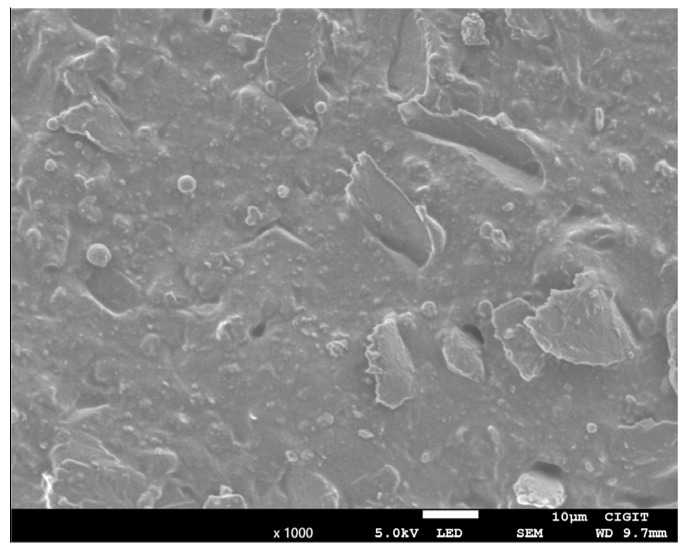
SEM diagram of the graphene and silicone rubber matrix.

Graphene’s aggregation problem is one of the primary factors limiting its widespread applicability. Therefore, an experiment was undertaken to see if the agglomeration issue arises when graphene is combined with silicone rubber. The specific steps were as follows: first, untreated graphene was mixed with the proper amount of silicone rubber; second, it was agitated with a mixer and the correct amount of curing agent was applied; it was evacuated with a vacuum pump; and finally, it was cured and molded at room temperature. Figure 2 demonstrates that after molding, the samples were examined using an electron microscope, which revealed that the untreated graphene could be well mixed with the silicone rubber. Consequently, the addition of graphene to silicone rubber does not produce agglomeration issues.

### 2.3. Resistance Creep Measurement

**Figure 3:**
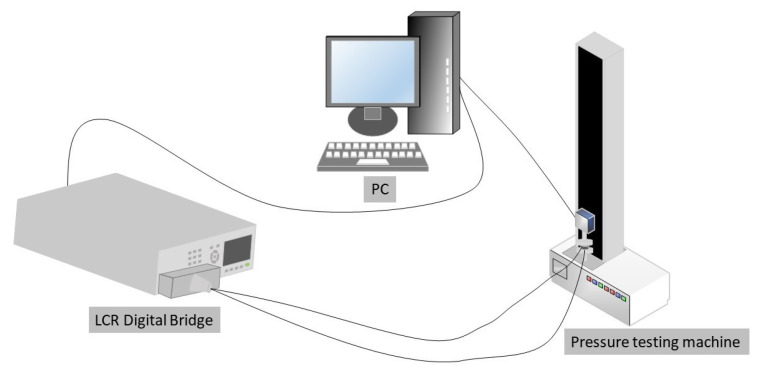
Resistance creep test system.

As depicted in Figure 3, the resistance creep test system included a press, a prepared sample, an LCR meter, and a computer. The prepared sample was encapsulated, and the sensing unit was positioned between two metal electrode plates. The program of the press controlled the process of delivering and releasing force, and 160 kPa of pressure was applied and sustained for 120 s. Throughout the procedure, the software measured the resistance in real time. To ensure complete contact between the electrode sheet and the polymer during this process, the pre-pressure was used to pre-pressure the electrode sheet, and the holding time was set at 120 s because the main creep phase appeared during this time, and the subsequent creep change was primarily the slow creep phase, which had a negligible impact on the overall creep size.

## 3. Results and Discussion

### 3.1. Mechanism of the Creep Behavior of Ni/MLG Particles on Polymers

**Figure 4:**
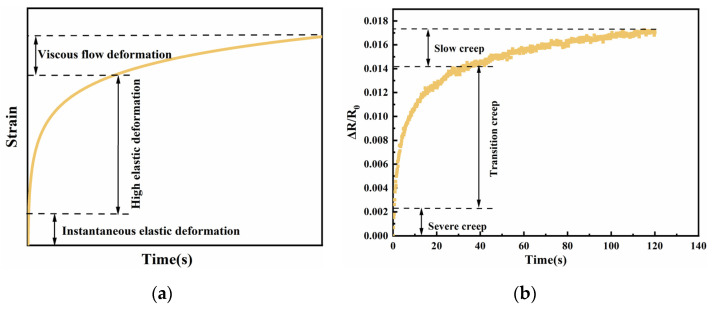
(**a**) Typical strain vs. time diagram at constant pressure. (**b**) Resistance change vs. time plot at 160 kPa constant pressure.

**Figure 5:**
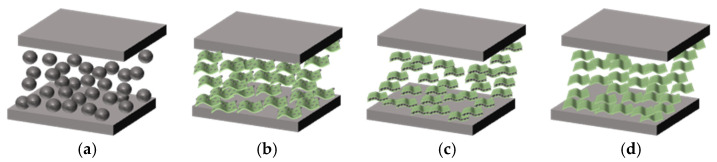
The main microscopic change process of nickel powder and graphene ratio. (**a**) Schematic diagram of the microstructure when nickel powder is filled alone. (**b**) Schematic diagram of the microstructure when nickel powder is in majority. (**c**) Schematic diagram of the microstructure when graphene is in majority. (**d**) Schematic diagram of the microstructure when graphene is filled alone.

The rule of polymer resistance creep is depicted in Figure 4b, and a comparison with Figure 4a demonstrates that the resistance creep process is remarkably similar to the strain creep process [12,25]. This suggests that, in the research of creep, evaluating the creep properties of materials by strain has the same effect as evaluating the creep characteristics of materials using electrical indicators. This is because a change in polymer resistance follows a change in strain. According to the quantum tunneling effect [26], strain induces a change in the tunneling effect, which impacts the resistance change. When an external force is applied to a polymer, its strain changes, which results in a change in resistance; the larger the force, the greater the change. If a certain force is applied to the sensing unit in a direction perpendicular to the polymer, it will cause the sensing unit to deform in that direction, which places the sensing unit in a compressed state, thereby reducing the particle spacing inside the sensing unit, which intensifies the quantum tunneling effect and ultimately reduces the sensing unit’s resistance. Therefore, based on the traditional strain creep process, the resistive creep process consists of three stages: a violent creep process, a transitional creep process, and a gradual creep process. Since the applied force does not exceed the yield strength of the material, the strain process’s rapid creep does not occur. The process is referred to as resistance creep failure The process of resistance change is characterized by a sudden shift in resistance at the moment of force application, a rapid decrease in resistance value, and the beginning of a gradual change when the resistance value rises dramatically for a brief period. As discussed previously, the fundamental source of the creep phenomenon is the poor molecular bonding between polymer chains, which produces slipping and tugging when a consistent force is applied. Consequently, the resistance to the creep of polymers can be enhanced by strengthening the links between polymers. Adding filled particles of varying dimensions to establish a stable microstructure in the polymer, boosting the polymer’s creep resistance, is an effective technique to strengthen the bond between polymers. This is illustrated in Figure 5.

Figure 5a depicts the polymer’s structure when only nickel powder was employed as a filler particle. The polymer resistance creep was primarily generated by the slip of the matrix and nickel powder, and the weak adhesion effect generated between the nickel powder and silicone rubber caused the slip of the matrix and nickel powder to be more severe when sufficient nickel powder content was added, resulting in a gradual decrease in the creep resistance of the polymer, and the decrease in nickel powder content resulted in a large resistance of the sensing unit.

When two distinct dimensions (nickel powder, graphene) of loaded particles are put into the sensing unit, there are three possible outcomes: the nickel powder is predominant, the nickel powder and graphene contents are equal, and the graphene content is predominant. As shown in Figure 5b, in the case where nickel powder constituted the majority of the material, the stable structure formed resembles two layers of nickel powder sandwiching a layer of graphene, and as the graphene content increases, this structure will become increasingly stable when subjected to forces. Because more nickel powder is required to form a stable structure with graphene, the next analysis was performed when the nickel powder content and graphene content were close to equal. When this occurs, the amount of this stable structure is greatly reduced, causing the polymer resistance to creep to be greater than in the case with less graphene. Lastly, examining the case of the graphene majority, as shown in Figure 5c, graphene plays a dominating role in the creep resistance of the sensing unit as the graphene content increases, and the creep resistance improves as the graphene content increases.

When there is only graphene or a very small amount of nickel powder in the sensing unit, as depicted in Figure 5d, its creep resistance becomes poor again because, as the content of nickel powder decreases, a very small amount of nickel powder cannot satisfy the combination with a large amount of graphene; it will cause the internal structure of the sensing unit to appear unbalanced.

### 3.2. Theoretical Model of Resistance Creep of MLG-NI-SR Copolymer

The Burgers model is a frequently used theoretical model for describing the creep behaviors of different materials. Combining the Maxwell model with the Kelvin–Voigt model in series results in a quadratic model. The Maxwell model consists of a linear spring connected in series to a Newtonian viscous pot, while the Kelvin–Voigt model consists of a linear spring connected in parallel to a Newtonian viscous pot [27,28].

Burger’s model divides the creep into three parts, which are transient elastic deformation, high elastic deformation, and viscous flow deformation. The transient elastic deformation can be replaced by the elastic part of the Maxwell model with an elastic modulus of *E*_1_; the high elastic deformation can be explained by the Kelvin model with an elastic modulus of *E*_2_ and a viscosity of $\eta_{2}$; the viscous flow deformation is explained by the viscous component of the Maxwell model with a viscosity of $\eta_{1}$ The specific equation is as follows:

(1) $$\varepsilon (t)=\frac{\sigma }{E_{1}}+\frac{\sigma }{E_{2}}[1-\exp(-\frac{E_{2}}{\eta_{2}}t)]+\frac{\sigma }{\eta_{1}}t$$

The relationship between strain and time *t* can be observed to be functional. Additionally, it corresponds with the three hypothesized resistance creep processes; however, it cannot be utilized to describe the rate of change of resistance versus time.

The tunnel effect theory is a conductivity theory based on the microscopic investigation of composite conductive polymer materials; according to the theory, the resistance of composite conductive polymer materials can be stated as [29,30]:

(2) $$R=\frac{L}{N}[\frac{8\pi \mathrm{hs}}{3a^{2}\gamma e^{2}}\exp(\gamma s)]$$

(3) $$\gamma =\frac{4\pi }{h}\sqrt{2m\varphi }$$

where *R* represents the resistance of the sensing unit, *L* represents the number of particles on a single effective conducting path, *N* represents the number of effective conducting paths in the sensing unit, h represents Planck’s constant, e represents the electron charge, *s* represents the minimum distance between particles in the sensing unit, a^2^ represents the effective cross-sectional area between two particles, m represents the electron mass and φ represents the potential barrier height.

In the compression process, it is assumed that the shortest distance between sensing unit particles is reduced from the initial *s* to *s*_1_ before the application of an external load, where *s*_1_ is dependent on the deformation of the sensing unit, and the deformation of each conducting particle is extremely small and negligible. Therefore, the relationship between the initial value of *s* and the reduced *s*_1_ of the shortest distance between the sensing unit particles may be calculated as:

(4) $$s_{1}=s(1+k\varepsilon )$$

where ε represents the strain of the sensing unit during compression, and *k* is a variable constant that can be interpreted as −1 during uniaxial compression. As the compression of the sensing unit causes the conductive path of the sensing unit to change, the relationship between the conductive path before the change and after the change is illustrated as follows [31]:

(5) $$N_{1}=N\exp(C_{1}\varepsilon +C_{2}\varepsilon^{2}+C_{3}\varepsilon^{3})$$

where *N* is the number of conductive paths at ε = 0, and *N*_1_ is the number of conductive paths after compression. After determining the relationship between the preceding equations, the relationship between the rate of change of resistance $\frac{R}{R_{0}}$ and the strain is produced by linking the following equations with them:

(6) $$\frac{\Delta R}{R_{0}}=1-\frac{\left(1-\varepsilon \right)}{\exp(C_{1}\varepsilon +C_{2}\varepsilon^{2}+C_{3}\varepsilon^{3})}$$

Combining the derived equation with Burger’s yields the rate of change of resistance $\frac{R}{R_{0}}$ vs. time *t*, i.e., the theoretical equation for the creeping resistance of the sensing unit:

(7) $$\frac{\Delta R}{R_{0}}=1-\frac{\left(1-f(t)\right)}{\exp(C_{1}f(t)+C_{2}f{\left(t\right)}^{2}+C_{3}f{\left(t\right)}^{3})}$$

where $R_{0}$ denotes the initial resistance, ΔR denotes the amount of change in resistance after compression by an external force, and f(t) is the previously described ε(t).

The aforementioned equation demonstrates that the rate of change of resistance is dependent on the elastic modulus and viscosity of the sensing unit, where the elastic modulus is primarily determined by the intermolecular bonding strength, and the viscosity is primarily determined by the intermolecular force of the liquid. The nickel powder, graphene content, and nickel powder-to-graphene ratio all influence the intermolecular bonding strength and force, which in turn influence the elastic modulus and viscosity of the sensing unit and eventually determine the sensing unit’s creep resistance. It can be seen that the creep resistance of the sensing unit is mainly determined by the nickel powder, graphene content, and the ratio of nickel powder to graphene. Therefore, these factors are fully discussed in the following.

### 3.3. Experimental Investigation of the Effect of Filler Particles on the Creep Resistance of Copolymers

#### 3.3.1. Effect of Nickel Powder on Polymer Resistance to Creep

**Figure 6:**
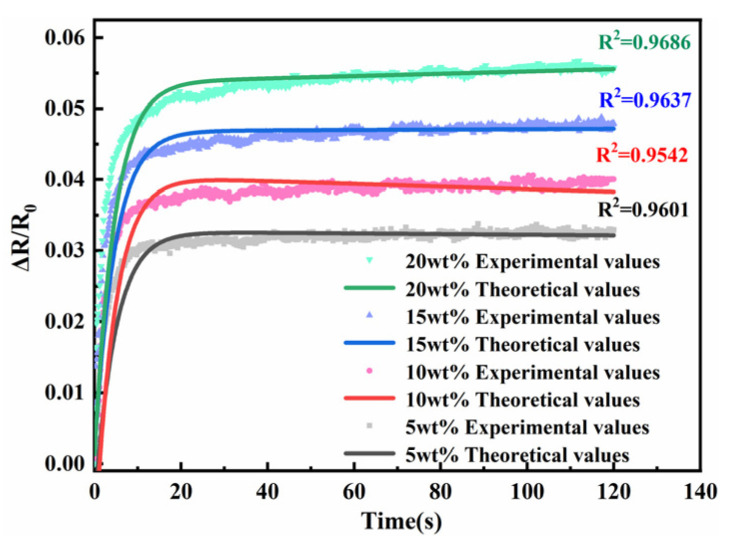
Diagram of nickel powder mass fraction vs. sensing unit resistance creep.

To determine the effect of nickel powder content on polymer resistance creep, samples with nickel powder content between 5 wt.% and 20 wt.% were prepared, and the theoretical and experimental values of the resistance creep model were compared, as shown in Figure 6. It can be seen that the increase in nickel powder content made the creep phenomenon of the sensing unit resistance more obvious. This is because the increase of nickel powder content leads to the movement of more filled particles during the application of pressure, which leads to the change of polymer resistance creep. The theoretical curves of the resistance creep model are in high agreement with the experimental values of the resistance creep at each mass fraction.

#### 3.3.2. Graphene’s Influence on Polymer Resistance to Creep

**Figure 7:**
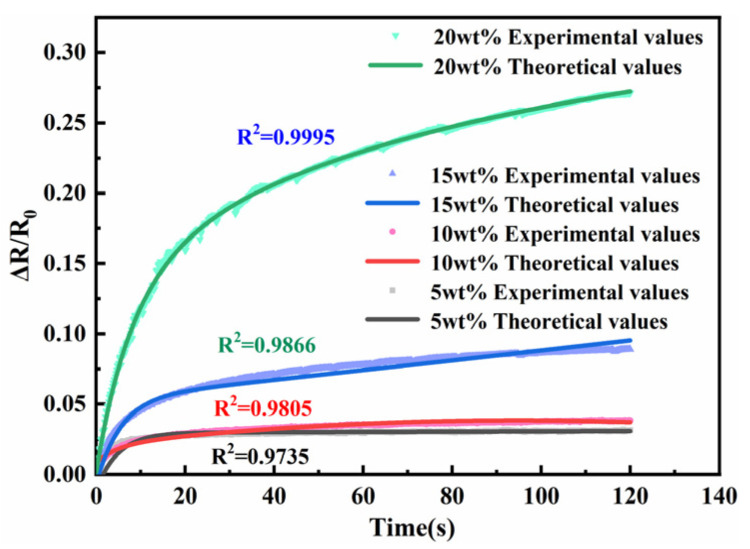
Diagram of graphene mass fraction vs. sensing unit resistance to creep.

To further investigate the effect of other filler particles on the polymer, the effect of graphene content on the resistance creep of the polymer is investigated in this section, as shown in Figure 7. From the figure, it can be seen that the effect of graphene content on the creep resistance of the polymer was similar to that of nickel powder, where the creep resistance gradually decreased with the increasing graphene content. The results in the above two subsections showed that both nickel powder and graphene content affect the creep of the sensing unit. This result is similar to the one obtained by Li et al. [32], that the proper adjustment of the influencing factor content can improve the sensing performance of the sensor as a way to obtain a better sensor. The comparison of the experimental values with the model theoretical values shows that the experimental values are in high agreement with the model theoretical values.

#### 3.3.3. The Influence of Nickel Powder and Graphene Ratios on the Creep Resistance of Polymers

The previous two subsections investigated the effect of polymer resistance creep in the case of nickel powder and graphene filling alone, respectively. To determine the magnitude of the effect of filling alone and compound filling on polymer resistance creep, the ratios of nickel powder and graphene in the polymer were investigated.

**Figure 8:**
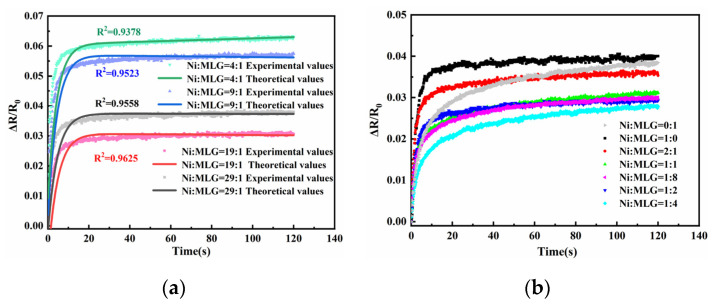
(**a**) Filling particle ratio vs. sensing unit resistance creep when the majority of the filling particles are nickel powder. (**b**) Filling particle ratio relative to sensing unit resistance creep when graphene constitutes the majority.

Figure 8 depicts the influence of nickel powder and graphene ratios on the creep resistance of polymer at 10 weight percent filled particles. Figure 8a demonstrates that the creep resistance is optimal when the nickel powder to graphene ratio is 19:1 and the nickel powder content is the majority. Figure 8b demonstrates that the resistance to creep is considerably improved when the ratio of nickel powder to graphene is 1:4. Comparing the two ratios revealed that the sensing unit has the highest creep resistance when the ratio of nickel powder to graphene is 1 to 4. This suggests that the performance of graphene in the majority is superior to that of nickel powder in the majority. Therefore, the next experiments were conducted with a nickel powder to graphene ratio of 1:4. Figure 8b demonstrates that, overall, the creep resistance created by adding simply nickel powder/graphene was inferior to that produced by the composite filling of nickel powder and graphene, and that the theoretical model is also applicable to the creep variation of resistance at different ratios.

**Figure 9:**
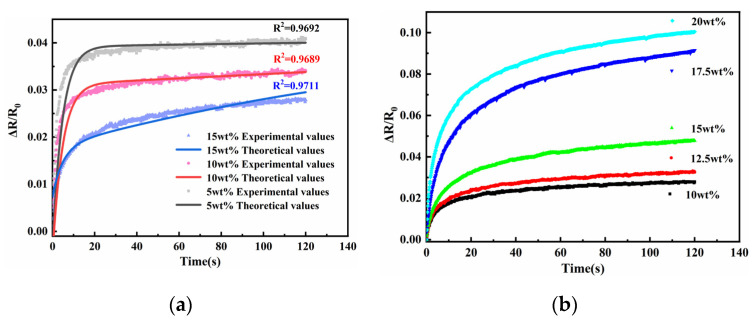
The effect of the mass fraction on the creep resistance of polymer at a constant ratio of nickel to graphene. (**a**) Small mass fraction resistance creep curve of the sensing unit. (**b**) Large mass fraction resistance creep curve of the sensing unit.

After determining the proportion of polymer-filled particles, the mass fraction of composite-filled particles was analyzed to determine the precise amount of filler particles; the findings are depicted in Figure 9. Figure 9a demonstrates that the creep resistance of the sensing unit improved gradually as the filler particle concentration increased from a low level to a high level. When the content of filler particles was further increased, it was observed that the creep resistance started to diminish, as illustrated in Figure 9b. Simultaneously, it can be observed that the creep resistance grew progressively less as the filled particle content increased. This is because the silicone rubber matrix does not affect the polymer’s resistance creep when the filler particle content is low. However, when the filler particle content is high, the silicone rubber matrix causes more drastic changes in the polymer’s resistance. When the amount of filled particles exceeds a given threshold, such as 10 wt.%, the total performance of the sensing unit is at its peak. In this instance, the creep between the filler particles and the silicone rubber matrix influences each other and reaches equilibrium when the sensing unit has the best performance against creep. When the concentration of the filler particles is raised, the creep resistance of the sensing unit drops proportionally. It can be shown that the mass fraction of filled particles had a bigger influence on the creep resistance of the sensing unit. In general, there is a synergistic effect between the particles, and this synergistic effect can effectively reduce the sensing unit creep. Li et al. [33,34] studied the hybridization of nanoparticles and also concluded that the interaction of multiple particles can effectively improve the mechanical properties of sensing units.

### 3.4. Experimental Investigation of the Effect of Filler Particles on the Creep Resistance of Copolymers

**Figure 10:**
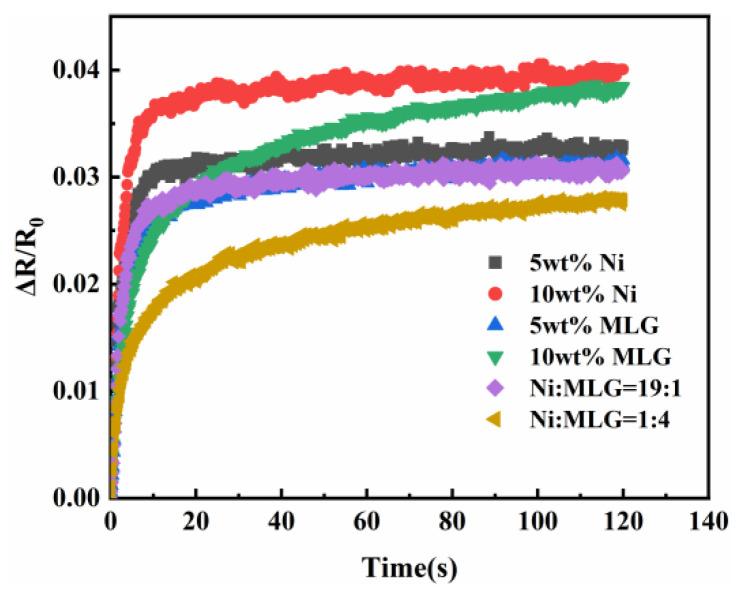
A comparison of the relative optimal resistance to creep for several affecting variables.

As stated previously, the effects of the polymer-filled particle mass fraction and two-filled particle ratios on the creep resistance of the polymer were investigated in this paper, and the relative optimal ratios were derived and compared under various influencing factors, as depicted in Figure 10, respectively. According to Figure 10, the creep resistance of the polymer filled with nickel powder alone was weaker than that of graphene alone, and this holds for either 5 wt.% or 10 wt.% of nickel powder or graphene, followed by a comparison of the ratios of nickel powder and graphene, which demonstrate that the creep resistance is stronger when graphene is the majority than when nickel powder is the majority. Comparing the strength of creep resistance between filling alone and two composite fillings revealed that the minimum creep value under composite filling is approximately 0.6 times the minimum creep value under filling alone with a constant mass fraction, indicating that composite filling can effectively mitigate the polymer resistance creep phenomenon.

To further determine the ratio of nickel powder to graphene, to examine the impact of stirring time on the sensing unit during the preparation process, and to optimize the ratios of the three parameters on the sensing unit of the haptic sensor, the ranges of graphene content between 8 wt.% and 8.9 wt.%, nickel powder content between 1.1 wt.% and 2 wt.%, and stirring time between 20 min and 60 min were selected.

This method of optimization uses the response surface method to carry out the optimization. The experimental scheme is designed first, then samples are fabricated and measurement indices relating to resistance creep are obtained using the experimental method. In this study, the resistance creep size is measured by the amount of resistance change when the pressure is held constant, as the resistance creep sizes may be compared by the amount of resistance change when the resistance value is generally constant. Table 2 below displays the specific outcomes.

The experimental model between resistance creep and the three components of nickel powder, graphene, and stirring time was determined by fitting experimental data with a multiple regression model.

(8) $$\begin{array}{l} Y=0.019+0.001A+0.0008B+0.0029C+\\ 0.0015AB+0.0002BC+0.0005A^{2}\\ +0.0006B^{2}-0.0001C^{2} \end{array}$$

where *Y* is the resistance creep size; *A* is the amount of nickel powder added; *B* is the amount of graphene added; *C* is the stirring time.

Table 3 displays the results of an ANOVA conducted using the response surface approach on Equation (8). The model’s *p*-value was less than 0.01, showing that the regression model was very significant; the misfit term was more than 0.05, suggesting that the model misfit was not significant, and the regression model had a high degree of fit. R^2^ equals 0.9622, indicating that the model had a high degree of correlation and predictive accuracy.

**Figure 11:**
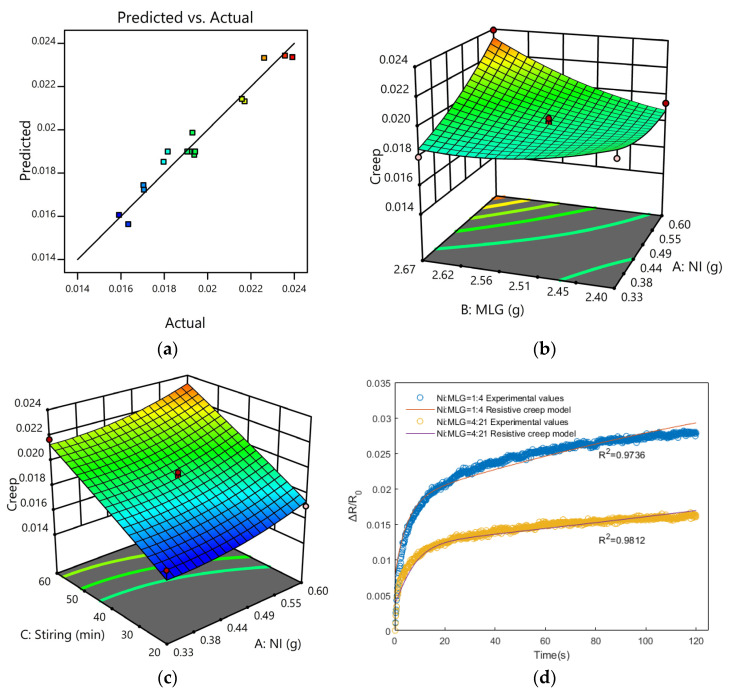
Graphs representing various optimization models: (**a**) experimental values and anticipated graphs; (**b**) nickel powder’s response surface to the influence of its interaction with graphene; (**c**) interaction effect response surface between stirring duration and nickel powder; (**d**) relationship between the observed values of creep for optimized ratios and pre-optimized ratios, on the one hand, and the theoretical values of the resistance creep model, on the other.

To further emphasize the high connection between the predicted and experimental values, the experimental values were compared to the predicted curves, and Figure 11a depicts the relationship between the experimental values and the predicted curves. It can be observed from the graph that the experimental and anticipated values had substantial linear overlaps.

Figure 11b,c depicts the 3D response surfaces of the interaction effects of nickel powder and graphene, stirring time, and nickel powder according to the regression model. The change of nickel powder and graphene content affect the resistance creep size, but it is not the case that more of both is better, but that the amount of both reaches the optimal amount to make the sensing unit produce the minimum resistance creep. In the case of constant nickel powder content, reducing the stirring time also improves the creep resistance of the sensing unit, and it can be seen from Figure 11c that the creep resistance performance was at its best when the stirring time was the shortest.

To further determine the optimized ratio of nickel powder and graphene, the parameters were optimized and compared with the experiments. The contents of nickel powder and graphene were controlled within the range of this experiment, and the stirring time was controlled within the specified range to measure the strength of the creep resistance of the sensing unit with the minimum change in resistance under this ratio. The regression model was solved to obtain the lowest optimum resistance to creep parameters as 0.44 g of nickel, 2.49 g of graphene, and 20 min of stirring.

To test the accuracy of the optimized formulation, the simulated values were compared to the actual measured values under the circumstances of 0.51 and 2.49 for nickel powder and graphene, respectively, to guarantee that the filled particle content was 10 wt.%. The simulation results indicate that the magnitude of resistance change was 0.0156342, while the experimental results indicate that the magnitude of resistance change was 0.0160219. The error rate between the simulation and the error value was 2.4%, indicating that the formulation obtained by simulation optimization was trustworthy. Then, by charting the relationship between the experimental value of creep resistance and the theoretical value of the model before and after optimization, the results indicate that the software-derived ratio was fair. Moreover, compared to the ratios before optimization, its resistance to creep decreased by approximately 45 percent.

Finally, to verify the reliability of the resistance creep model, as depicted in Figure 11d, the theoretical values of the model were compared with the experimental values of resistance creep before and after optimization, and it was concluded that the theoretical values of resistance creep were highly close to the experimental values. Their R^2^ were 0.9736 and 0.9812, respectively, indicating that the proposed resistance creep model is reliable and can be effectively used to predict resistance creep.

## 4. Conclusions

In this work, we effectively provided a strategy to reduce the creep of polymer resistance. Additionally, we built a creep model between the rate of change of resistance and the pressure, and we came to the following conclusions as a result:

(1) A relationship was established between the electrical creep and the mechanical creep of polymer nanocomposites, and a resistive creep model was developed as a result of these findings.
(2) The effective reduction of sensing unit creep was experimentally verified by compounding filled particles of varying nature and structure. The best effect of reducing polymer creep was obtained when the mass fraction of compounded filled particles was 10% and the ratio of nickel powder to graphene was between 19:1–9:1 and 1:4–1:8. This combination produced the best results in terms of reducing polymer creep.
(3) The approach of response surface optimization was used to acquire the optimal ratios. When compared to the resistance creep before optimization, it was lowered by around 45%, while it was decreased by approximately 60% when compared to the resistance creep of the single-component filling.
(4) The theoretical values of the model were compared with the experimental values of the ratios before and after optimization. The R^2^ value between the model and the experimental values was 0.9736 and 0.9812, respectively, which indicates that the suggested theoretical model is dependable and correct.

## Figures and Tables

**Table 1:** Sensing unit preparation sample parameters.

Group	Quality Fraction (wt.%)	Ni and MLG Ratio	SR and Silicone Oil Ratio
1	5, 10, 15, 20	1:0	10:1
2	5, 10, 15	0:1	10:1
3	10	1:0, 29:1, 19:1, 9:1, 4:1	10:1
4	10	1:0, 2:1, 1:1, 1:2, 1:4, 0:1	10:1
5	5, 7.5, 10, 12.5, 15, 17.5, 20	1:4	10:1

**Table 2:** Orthogonal experimental design and results.

Group	Influencing Factors			Resistance Creep
	Ni (g)	MLG (g)	Mixing Time (min)	
1	0.33	2.535	60	0.021712
2	0.465	2.535	40	0.019429
3	0.6	2.67	40	0.023916
4	0.6	2.4	40	0.019388
5	0.465	2.535	40	0.019122
6	0.465	2.4	20	0.015916
7	0.33	2.535	20	0.016341
8	0.6	2.535	20	0.017043
9	0.33	2.67	40	0.017972
10	0.6	2.535	60	0.022612
11	0.465	2.67	20	0.017065
12	0.33	2.4	40	0.019304
13	0.465	2.535	40	0.018163
14	0.465	2.535	40	0.019066
15	0.465	2.67	60	0.023573
16	0.465	2.535	40	0.019177
17	0.465	2.4	60	0.021585

**Table 3:** ANOVA examination of the experimental response surface.

Source	Sum of Squares	Degrees of Freedom	Mean Square	*p*-Value	
Model	0.0001	9	0.0001	0.0004	
Lack of Fit	2.64 × 10^−6^	3	8.8 × 10^−7^	0.1163	
Pure Error	3.08 × 10^−6^	4	2.34 × 10^−7^		
R^2^					0.9622

## Data Availability

The data presented in this study are available in this article.
